# ASO Author Reflections: Surgical Management of Paratesticular Rhabdomyosarcoma

**DOI:** 10.1245/s10434-024-15687-x

**Published:** 2024-06-29

**Authors:** Illya Martynov, Monika Sparber-Sauer, Amadeus Heinz, M. Christian Vokuhl, Martin Ebinger, Jens Gesche, Marc Münter, Ewa Koscielniak, Jörg Fuchs, Guido Seitz, Cooperative Soft Tissue Sarcoma Study Group

**Affiliations:** 1 Department of Pediatric Surgery and Urology, University Hospital Giessen-Marburg, Philipps-University, Marburg, Germany; 2 Department of Pediatric Surgery, University Hospital Giessen-Marburg, Giessen, Germany; 3 Pädiatrie 5 (Pädiatrische Onkologie, Hämatologie, Immunologie), Klinikum der Landeshauptstadt Stuttgart gKAöR, Olgahospital, Stuttgart Cancer Center, Zentrum für Kinder-, Jugend- und Frauenmedizin, Stuttgart, Germany; 4 University of Medicine Tübingen, Tübingen, Germany; 5 Section of Pediatric Pathology, Department of Pathology, University Hospital Bonn, Bonn, Germany; 6 Department of Pediatric Hematology and Oncology, University Children’s Hospital, Tuebingen, Germany; 7 Pediatric Surgery, Josefinum, Augsburg, Germany; 8 Department of Radiooncology, Klinikum Stuttgart, Stuttgart, Germany; 9 Department of Pediatric Surgery and Urology, University Children’s Hospital, Tuebingen, Germany

## Past

Paratesticular rhabdomyosarcoma (PTRMS) is the most prevalent non-germ-cell scrotal malignant tumor, accounting for approximately 5% of all pediatric scrotal masses and 7% of all pediatric rhabdomyosarcomas (RMS).^1,2^ The recommended surgical approach for PTRMS involves primary inguinal orchidectomy with high ligation of the spermatic cord before tumor mobilization. However, protocol violations during primary surgery are common and often lead to incomplete tumor resections, necessitating revisional surgeries known as pretreatment re-excision (PRE). PRE can downstage tumors from IRS III (macroscopically incomplete tumor resection, R2) or IRS II (microscopically incomplete tumor resection, R1) to IRS I (microscopically complete tumor resection, R0), thus achieving excellent oncological outcomes. Nevertheless, there is a paucity of data on the effects of unsuccessful PRE, which may result in local R1/R2 situation, on event-free survival (EFS) or overall survival (OS).

## Present

We evaluated the impact of the quality of initial and subsequent surgeries on the survival of patients with non-metastatic and metastatic PTRMS enrolled in the Cooperative Weichteilsarkom Studiengruppe (CWS) trials (CWS-96, 2002P) and the Soft Tissue Sarcoma Registry (SoTiSaR). Our findings reveal that the quality of local surgical control is frequently unsatisfactory, characterized by a high incidence of protocol violations during primary surgery (42%) and PRE (20%).^3^ Further, our analysis, using an unadjusted log-rank test, highlighted that R status following PRE is a significant prognostic factor for 5-years OS. This underscores the critical need for strict adherence to surgical guidelines, particularly during revisional surgery.

## Future

Our study indicates that improvements are necessary in the quality of surgical local control for PTRMS. The path forward should focus on enhancing surgical training and preoperative planning to reduce protocol violations during both primary surgery and PRE. Notably, an R1 resection following PRE was associated with decreased OS, suggesting its potential role as a prognostic factor for patients with localized PTRMS.
